# Serotype Diversity and Molecular Characterization of Foot-and-Mouth Disease Viruses From Outbreaks in Ethiopia (2019–2023): Re-Emergence of SAT 2 After 30 Years

**DOI:** 10.1155/tbed/6670343

**Published:** 2025-11-26

**Authors:** Daniel Gizaw, Bayeta Senbata, Aynalem Fentie, Tsion Bilata, Demessa Negessu, Ayelech Muluneh, Dereje Shegu, Hagos Ashenafi, Nick J. Knowles, Jemma Wadsworth, Valerie Mioulet, Hayley M. Hicks, Mengistu Legesse, Tesfu Kassa, Donald P. King

**Affiliations:** ^1^Animal Health Institute (AHI), Sebeta, Ethiopia; ^2^Aklilu Lemma Institute of Health Research, Center for Pathobiology Research, Addis Ababa, Ethiopia; ^3^World Reference Laboratory Foot-and-Mouth Disease (WRLFMD), The Pirbright Institute, Pirbright, UK

**Keywords:** cattle, distribution, diversity, Ethiopia, FMD virus, serotypes

## Abstract

Ethiopia faces significant economic losses from foot-and-mouth disease (FMD). Laboratory diagnostic tools such as antigen detection ELISA (Ag-ELISA), reverse transcription polymerase chain reaction (RT-PCR), and sequencing provide important information that underpin control initiatives. In this study, 411 samples (275 epithelial tissues, 12 oropharyngeal probang cup scrapings and fluids, 122 swab samples, and two whole blood) collected from cattle with clinical signs of FMD were tested to determine serotype diversity of the FMD viruses (FMDVs) present in Ethiopia during a 5-year period (2019–2023). RT-PCR testing showed that most samples, 81.1% (172/212) were positive for FMDV genome while 48.2% (198/411) of the samples were positive for FMDV antigen using ELISA, identifying serotypes O (10.9%), A (7.3%), Southern African Territories (SAT) 1 (1.7%), and SAT 2 (19.5%). Furthermore, evidence for mixed serotype infection was observed for 36 samples using the Ag-ELISA. Viral protein (VP) 1 sequencing for FMDV was performed on 94 samples, confirming the presence of three FMDV serotypes (O, A, and SAT 2). There was no molecular sequence evidence for outbreaks due to SAT 1 during this period, suggesting that the Ag-ELISA results for this serotype may have been false positives. Together with the Ag-ELISA data, the sequences highlighted a dramatic increase in the dominance of serotype SAT 2 viruses during the period of the study, associated with outbreaks due to the emerging SAT2/XIV topotype after a period of absence of more than 30 years. These data highlight Ethiopia's dynamic FMD landscape, informing national and regional control. These findings are crucial for understanding FMDV in Ethiopia and vaccine selection, although more geographically broad and sequencing-intensive studies may be needed to define a more comprehensive understanding of the national disease epidemiology.

## 1. Introduction

Ethiopia has a livestock population of more than 60 million cattle, 31 million sheep, and 32 million goats [1]. These livestock are estimated to contribute 37%–87% of household incomes, 35%–49% of the agricultural gross domestic product (GDP), and 15%–17% of the national GDP [2]. Foot-and-mouth disease (FMD) is a highly infectious disease that can impact upon the productivity of cloven-hoofed animals. The disease is characterized by fever, anorexia, excessive salivation, and the appearance of vesicular blisters on the tongue, nose, mouth, teats, snout and feet, which can cause food abstinence and lameness [3]. The disease is caused by a virus FMD virus (FMDV) that belongs to the genus *Aphthovirus* of the family Picornaviridae [4] and consists of seven immunologically distinct serotypes (O, A, C, Asia 1, Southern African territories [SATs] 1, SAT 2, and SAT 3) [5]. FMDV has a single-stranded, positive-sense RNA genome of about 8500 nt in length and is enclosed by an icosahedral capsid [6, 7]. FMD is widely reported in Africa and Asia, particularly in areas with high levels of poverty and livestock density, where it causes significant economic losses [8, 9]. The impacts of FMD extend beyond individual herds and farmers to affect the national economy [10–12]. A previous study in Ethiopia estimated average losses at $76 per affected herd in mixed crop-livestock systems and $174 in pastoral systems due to costs for diagnostics, vaccination, control, and restriction on trade [10, 13, 14]. Preventing and controlling FMD involving detection with supporting diagnostics is a crucial step to enable Ethiopian farmers to access global livestock markets [15, 16].

The epidemiology of FMD in East Africa (EA) is complex, characterized by the presence of five FMDV serotypes (O, A, SAT 1, SAT 2, and SAT 3). FMD is endemic in Ethiopia, where three FMDV serotypes (O, SAT 2, and A) are the most prevalent [17]. Serotype O FMDVs are most frequently detected and are classified within the EA-3 and EA-4 topotypes [18, 19]. Circulating serotype A FMDVs include genotypes G-I, G-IV, and G-VII of the AFRICA topotype [19]. Serotype SAT 2 was first recorded in Ethiopia in 1989–1991 [18, 20] and was later detected between 2007 and 2023. Outbreaks due to SAT 2 in Ethiopia have been caused by topotypes IV, XIV, and XIII [18–20]. The SAT 2 topotype IV has also occurred in Kenya, Tanzania, Uganda, Malawi, and Burundi [18, 19]. More distantly related viruses belonging to this topotype were also found in Sudan [18, 19, 21]. The SAT 1 topotype IX only includes viruses from Ethiopia, detected in the Benchi-Maji zone during 2007–2008 [22] and despite frequent serological evidence, no SAT 1 viruses have been isolated since 2007 [3].

Ethiopia's numerous FMD serotypes and susceptible livestock/wildlife populations create complex temporal and spatial outbreak patterns [23]. FMD outbreaks occur throughout the year and impact the whole country [18, 19, 24, 25]. A temporospatial study of FMD outbreaks (2010–2019) in Ethiopia reported 397,735 cases in 1423 outbreaks across all regional states of Ethiopia [23]. However, only an annual average of 247 outbreaks were reported from export sourcing areas of southeastern Ethiopia [26] over the same period. Therefore, although high numbers of deaths sometimes occur in livestock due to FMD [27], under reporting by farmers due to the disease's endemic nature and lack of incentives means the burden of disease is not reflected by the number of cases and outbreaks that are officially reported [27, 28]. The FMDV is constantly evolving, and it is therefore important to understand the molecular epidemiology of the virus to understand risk pathways, monitor the spread of FMDV, and ensure that appropriate vaccines are selected to control field outbreaks. Ethiopia is at stage 1 of the progressive control pathway (PCP) [27], and a risk-based strategic plan (RBSP) for controlling FMD is required to reduce FMDV circulation to progress to the next stage [16]. The objective of this study was to characterize FMDVs circulating in Ethiopia from 2019 to 2023, identifying serotypes, topotypes, and genetic variants to inform effective monitoring and response strategies for managing and mitigating the impact of FMDV on livestock populations.

## 2. Materials and Methods

### 2.1. Study Area and Sample Collection

Outbreak investigations were conducted for suspected FMD outbreaks that occurred across Ethiopia between 2019 and 2023. A total of 411 samples were collected from 11 regional states and two administrative cities. During the outbreaks, investigations were conducted on farms reporting cases of FMD in cattle, goats, and sheep to document clinical signs and details of the production system, including the numbers of at-risk animals. The specimens collected consisted of 12 oropharyngeal probang cup scrapings and fluids, 122 swab samples, 275 epithelial tissues, and two whole blood samples. Each sample was uniquely labeled, stored on an ice pack, and immediately transported to the Animal Health Institute (AHI).

### 2.2. Virus Isolation

For virus isolation, samples were excised from transport medium (an equal volume mixture of glycerol and 0.04 M phosphate buffer). A 10% (w/v) epithelial suspension was prepared by mechanical disruption with sterile sand using a pestle and mortar, followed by centrifugation for clarification. The sample was then centrifuged at 3000 x *g* for 30 min and subsequently filtered using a 0.45 µm syringe filter. Confluent monolayers of BHK-21 or goat tongue cell lines (ZZ-R) were infected with a 0.5 mL tissue suspension and spread over a cell sheet in a 25 cm^2^ tissue culture flask by rocking for 30 min to enhance adsorption. Following this, 10 mL of minimum essential medium (MEM) with supplements and antibiotics was added, and the flasks were incubated at 37°C and 5% CO_2_. Cells were examined twice daily for cytopathic effect (CPE), which appeared as early as 12 h postinfection. Infectious fluid was harvested for molecular analysis at 48–72 h [29, 30]. When no CPE was observed, blind passages were performed to maximize the chances of virus isolation and identification [30]. The virus was then characterized by antigen detection, ELISA, or sequencing to understand the molecular epidemiology of the viruses detected.

### 2.3. Antigen Detection ELISA (Ag-ELISA)

FMDV serotyping was performed using, an Ag-ELISA (IZSLER, Brescia, Italy), following the manufacturer's instructions. Each plate included positive and negative controls for seven serotypes (O, A, C, Asia 1, SAT 1, and SAT 2) and a pan-FMDV control. The process involved sample/buffer addition, incubation, washing (four cycles), conjugate addition (conjugate A to rows A-F, conjugate B to G-H), a second incubation, another four washing cycles, substrate/chromogen addition, a dark incubation, reaction stopping, and optical density (OD) measurement at 450 nm. Interpretation of the test validity and outcome criteria for the samples followed the manufacturer's instructions included with the ISZLER ELISA kits (Brescia, Italy).

### 2.4. Molecular Detection Using Reverse Transcription Polymerase Chain Reaction (RT-PCR)

QIAamp Viral RNA Extraction Kit (QIAGEN) was used following the manufacturer's protocol. Universal primer sets designed to target conserved regions of the FMDV genome were used in RT-PCR assays for the detection of all seven FMDV serotypes. The extracted RNA was subjected to RT-PCR with these primers: forward primer (FMDV 3DF): 5′-ACTGGGTTTTACAAACCTGTGA-3′, reverse primer (FMDV 3DR): 5′-GCGAGTCCTGCCACGGA-3′, and probe (FMDV Probe 3DP): 5′-(6FAM) TCCCTTTGCACGCCGTGGGAC (TAM)-3′ using the QIAGEN One-Step RT-PCR Kit under these conditions: 50°C for 30 min; 95°C for 15 min; 35 cycles of 94°C for 30 s, 55°C for 30 s, and 72°C for 90s; and a final 72°C for 10 min (Rotor-Gene Q thermal cycler, Qiagen, Germany) [31]. This protocol enabled RT-PCR amplification and detection of the FMDV 3Dpol gene [32–35]. Target amplification was confirmed by the presence of a curve and a cycle threshold (Ct) value, determined by SDS software above background fluorescence. Amplification with Ct values less than 35.0 was judged positive, whereas amplification with no Ct values was considered negative. RT-PCR results defined samples as either FMDV genome detected or no virus detected (NVD).

### 2.5. Sequencing of Viral Protein (VP) 1 of FMDV

VP1 sequencing was attempted for 148 representative samples that were sent to the FAO World Reference Laboratory for FMD (WRLFMD) based at the Pirbright Institute (United Kingdom). RNeasy Mini kits (Qiagen, Crawley, West Sussex, UK) were used to extract total RNA from the virus isolates, and one-step RT-PCR kits (Qiagen) were used to amplify the VP1 region of FMDV. The oligonucleotide primers used were as follows: O-1C244F/EUR-2B52R; FMD-3161F/FMD-4303R for serotype O; A-1C562F/EUR-2B52R; FMD-3161F/FMD-4303R for serotype A; and SAT2-1C445F/SAT2-P1-1223F and SAT-2B208R for serotype SAT 2 [5] using amplification conditions previously described [5]. The amplicons were sequenced using the BigDye Terminator v3.1 Cycle Sequencing kit (Applied Biosystems, Carlsbad, USA) with the following oligonucleotides: NK72 for all serotypes; O-1D296F, O-1C583F and O-1D628R for serotype O; A-1C612F for serotype A; and SAT2-1C445F; SAT2-P1-1223F; SAT2VP3-AB/SAT-2B208R and SAT2-D for serotype SAT 2 [5]. An ABI 3730 DNA Analyser (Applied Biosystems, Foster City, USA) was used for DNA sequencing. Nucleotide sequences covering the complete VP1 coding region were assembled from at least one forward and one reverse read for each sample using SeqMan Pro (Lasergene 15.0 software; DNAStar Inc., Madison, WI, USA).

### 2.6. Phylogenetic Analysis

Complete VP1-coding nucleotide sequences were aligned using MEGA 12 (ClustalW codon alignment option) [36]. Using the maximum-likelihood (ML) method of phylogenetic tree construction with 500 bootstrap replicates using adaptive bootstrapping. Length of the FMDV VP1 coding regions for serotypes O, A, and SAT 2 were 642, 639, and 648 nucleotides, respectively (these data included six nucleotides of the 2A region). ML trees for each of the serotype alignments were reconstructed using MEGA 12 software. Nucleotide substitution models with the lowest BIC for each serotype were determined in MEGA 12; they were TN93+I (type O), HKY + G (type A), and TN3+G+I (type SAT 2). The resulting ML trees classified FMDV into geographically restricted clusters known as topotypes, as described [6, 37].

## 3. Results

### 3.1. Outbreak Distribution

**Figure 1:**
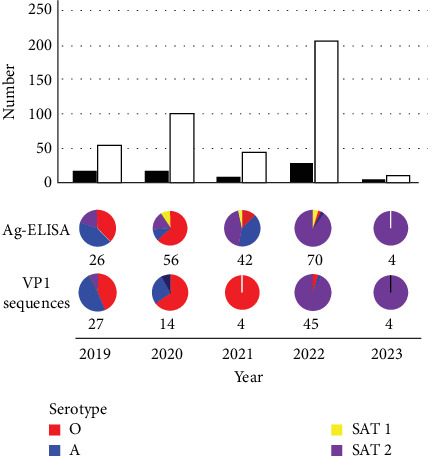
Temporal distribution of FMD outbreaks (black bars) and corresponding samples (open bars) across the five years of the study. FMDV serotypes detected by Ag-ELISA and VP1 sequencing are represented in the pie-charts (only FMDV positive results are shown), which denote the number of samples tested in each year.

The numbers of FMD outbreaks varied across the different regions of Ethiopia over the 5 years of the study (Figure 1). A total of 411 samples were processed (from Oromia [*n* = 254], Addis Ababa [*n* = 4], Afar [*n* = 24], Amhara [*n* = 34], Benishangul-Gumuz [*n* = 14], Southern Nations, Nationalities and Peoples' Regional State [*n* = 54), Somali [*n* = 9], and Tigray [*n* = 18]. FMD outbreaks were reported every year within the study period and were distributed mainly throughout the central and western regions of the country.

The limited availability of RT-PCR reagents at the AHI posed a challenge to the scope of this study, preventing the analysis of the entire sample set by molecular methods. As a result, only 212/411 of the collected samples were tested using RT-PCR. These analyses revealed that 81.1% (172/212) of the samples were positive for FMDV genome (3D target), while 18.9% (40/212) generated negative results with the RT-PCR (Table 1).

These RT-PCR tested samples were also tested using Ag ELISA as described in Table 2. The 132 samples were both positive and 11 samples were both negative. Based on the results, the Ag-ELISA test exhibited a sensitivity of 77% (132/172), correctly identifying most true positive cases. However, its specificity was notably poor at 28%, indicating a high frequency of false positive results. The test's positive predictive value (PPV) was 82% (132/161), confirming that a positive result is a strong indicator of a true positive. Conversely, the negative predictive value (NPV) was only 22% (11/51), rendering a negative result from the test largely unreliable. With an overall accuracy of 67% (143/212), the RT-PCR appears useful for initial screening.

Out of the total 411 samples, FMDV antigen was detected in 198 samples (48.2%) using Ag-ELISA (serotypes O, A, SAT 1, and SAT 2) across different regions, as described in Table 3. Of the serotypes detected, 45 (10.9%) were positive for FMDV serotype O, 30 (7.3%) for serotype A, 7 (1.7%) for serotype SAT 1, and 80 (19.5%) for serotype SAT 2. Evidence for mixed infections comprising more than one serotype was found for 36 samples, specifically serotypes SAT 2 and SAT 1 (Table 3).

Most regions in Ethiopia experienced outbreaks due to more than one serotype during the study period (Table 3), except for Addis Ababa and Afar, from which only a single serotype was detected (O and SAT 2, respectively). FMDV serotype SAT 2 was the most frequently detected serotype identified by Ag-ELISA, followed by FMDV serotype O. Notably, SAT 2 was detected for the first time in the eastern region of Afar, but was not found in Somalia during this study. To our knowledge, no SAT viruses were previously detected in the eastern parts of Ethiopia, including Somali, Afar, and Eastern Oromia. Evidence for serotype SAT 1 using Ag-ELISA was only found for seven samples. The Ag-ELISA test kit used could detect FMD serotypes C and Asia 1, but neither of these serotypes was detected in any of the samples. Serotype C has not been detected in Ethiopia since 1984, while Asia 1 has never been detected even in Africa [38].

Mixed serotype infections were detected with the Ag-ELISA. Specifically, coinfections with FMDV O and SAT 2, as well as FMDV A and SAT 2, were observed. Additionally, samples identified as positive for SAT 2 showed evidence of mixed infection with SAT 1, as described in Table 3.

**Figure 2:**
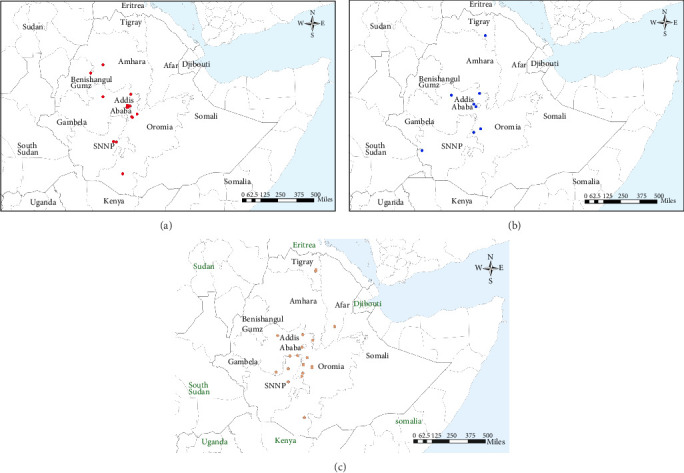
The geographical distribution of FMDV: (A) serotype O (EA-3 and EA-4) (red coloured dot), (B) Serotype A (A/AFRICA/G-IV), (blue coloured dot), and (C) serotype SAT 2 (VII/Alx-12, VIII/Lib-12, XIII, and XIV) (orange coloured dot).

The distribution of FMDV serotypes fluctuated annually, demonstrating the dynamic nature of FMD in the country. While serotype A was the most prevalent in 2019, the serotype profile changed in 2020, with serotype O becoming more dominant before the expansion of serotype SAT 2 from 2021 to 23. (Figure 2). The outbreaks due to serotype SAT 2 were widely distributed in Ethiopia.

### 3.2. VP1-Coding Sequencing

Phylogenetic analysis was used to establish the genetic relationship between viruses that caused Ethiopian outbreaks and viruses detected from the neighboring countries. VP1 sequencing was successful for 94/148 (63.5%) of samples sent to the WRLFMD corresponding to those samples that were positive by RT-PCR when tested in the UK. These samples originated from a wide array of geographical locations across the country, specifically areas where outbreaks of FMD had been officially documented during the 5-year duration of this study (Figure 2). Genetic sequencing of these FMDV samples identified the presence of distinct serotypes, namely O, A, and SAT 2, each exhibiting genetic variation that could be used to allocate the samples to different topotypes.

The predominant serotype detected among the 94 samples was FMDV SAT 2 (*n* = 49), followed by FMDV O (*n* = 27) and FMDV A (*n* = 18) as detailed in (Supporting Information 1: Table S1). Phylogenetic trees showed how FMDV was dispersed within Ethiopia and between neighboring countries.

### 3.3. FMDV Serotype O

**Figure 3:**
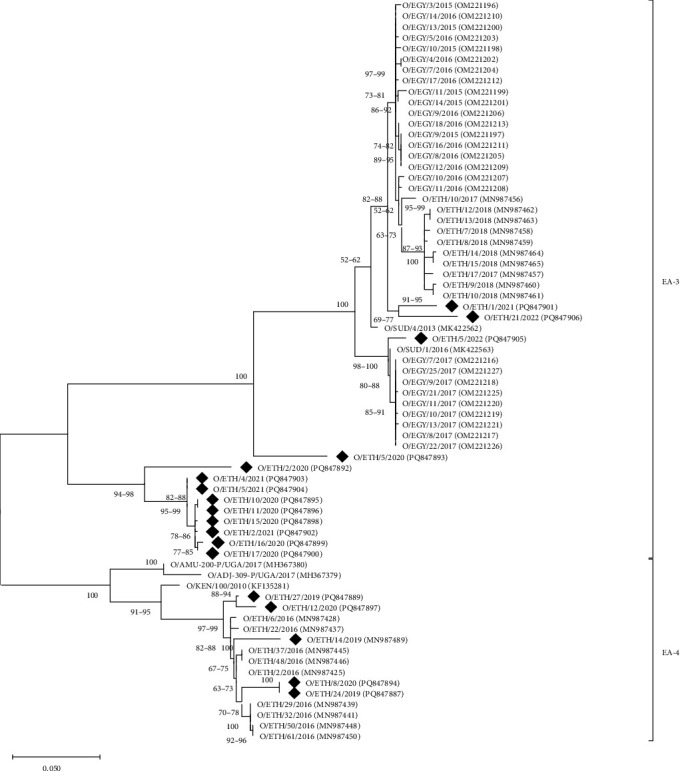
A midpoint-rooted maximum likelihood tree of the VP1-coding region of serotype O FMD viruses detected in Ethiopia (indicated by black diamonds). The tree was produced using MEGA 12 [36] using a multiple sequence alignment. The phylogeny was inferred using the maximum likelihood method and Tamura (1992) model of nucleotide substitutions, and the tree with the highest log likelihood (−3,234.42) is shown. The percentage of replicate trees in which the associated taxa clustered together, where the number of replicates (107) was determined adaptively [36], is shown next to the branches. The rate model allowed for 58.19% of sites to be evolutionarily invariable (I). The analytical procedure encompassed 69 nucleotide sequences with 642 positions in the final dataset.

The VP1 sequence analysis of serotype O revealed the presence of two East African topotypes, EA-3 (*n* = 20) and EA-4 (*n* = 7), in samples collected from 2019 to 2023 (Figure 3). FMDV topotype O/EA-3 viruses were reported every year from 2019 to 2023, except for 2023, and were most closely related to viruses from previous years in Ethiopia, Sudan, and Egypt. FMDV topotype O/EA-4 was reported only in 2019 and 2020, although these sequences provided evidence for the long-term maintenance of this topotype within the country (since at least from 2016).

### 3.4. FMDV Serotype A

**Figure 4:**
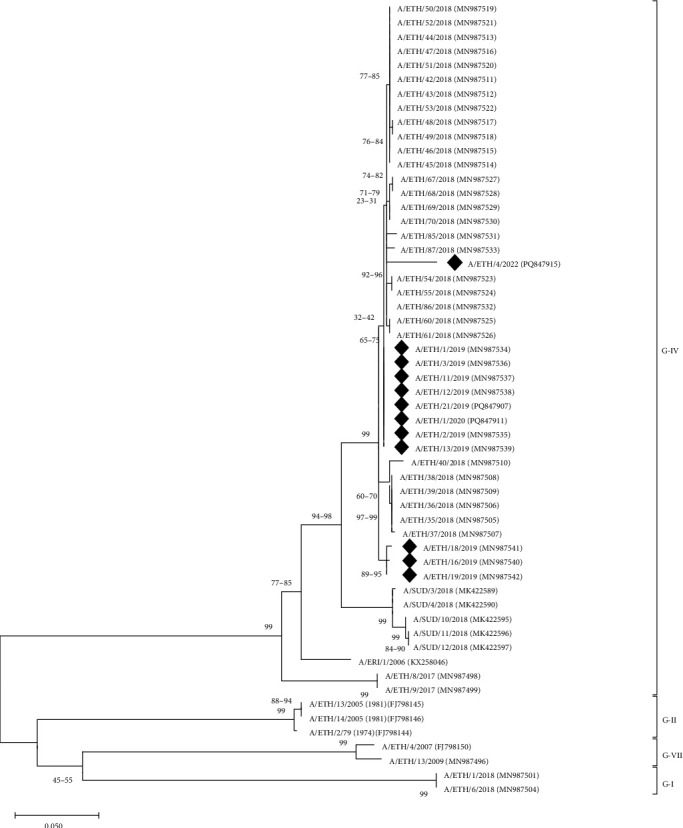
A midpoint-rooted maximum likelihood tree of the VP1-coding region of serotype A FMD viruses detected in Ethiopia (indicated by black diamonds). The tree was produced using MEGA 12 [36] using a multiple sequence alignment. The phylogeny was inferred using the maximum likelihood method and Hasegawa-Kishino-Yano (1985) model of nucleotide substitutions, and the tree with the highest log likelihood (−2814.92) is shown. The percentage of replicate trees in which the associated taxa clustered together, where the number of replicates (101) was determined adaptively [36], is shown next to the branches. The evolutionary rate differences among sites were modeled using a discrete Gamma distribution across five categories (+G, parameter = 0.3289). The analytical procedure encompassed 56 nucleotide sequences with 639 positions in the final dataset.

The VP1 sequences revealed only topotype A/AFRICA/G-IV (*n* = 18) from 2019, 2020, and 2022 (Figure 4). FMDV A/AFRICA/G-IV lineage sequences collected in this study comprised a monophyletic clade that comprised sequences only from Ethiopia (2018, 2019, 2020, and 2022), which was most closely related to VP1-coding sequences from other countries in EA (Sudan, 2018, and Eritrea, 2006).

### 3.5. FMDV SAT 2

**Figure 5:**
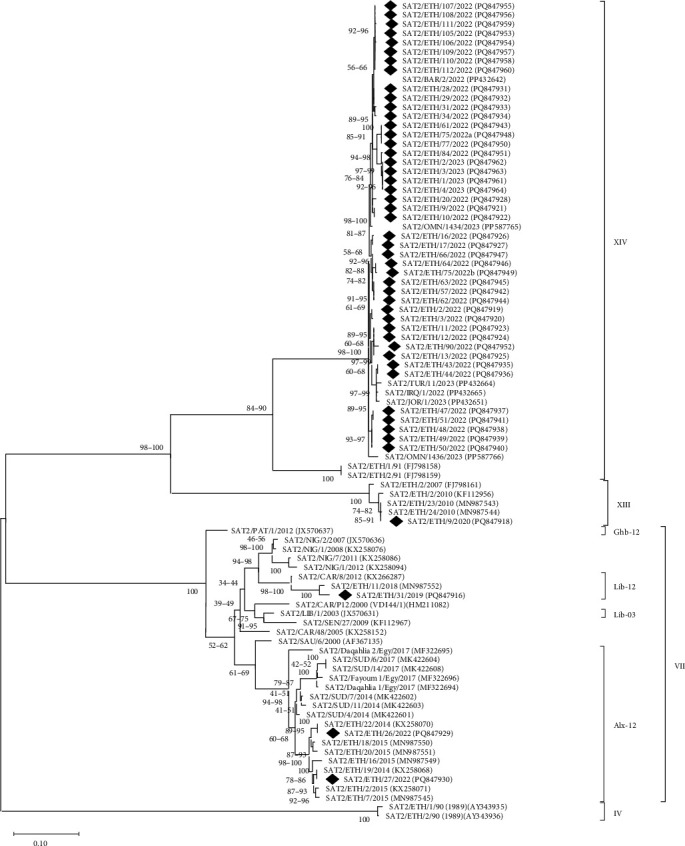
A midpoint-rooted maximum likelihood tree of the VP1-coding region of serotype SAT 2 FMD viruses detected in Ethiopia (indicated by black diamonds). The tree was produced using MEGA 12 [36] using a multiple sequence alignment. The phylogeny was inferred using the maximum likelihood method and Tamura-Nei (1993) model of nucleotide substitutions and the tree with the highest log likelihood (−5567.69) is shown. The percentage of replicate trees in which the associated taxa clustered together, where the number of replicates (114) was determined adaptively [36], is shown next to the branches. The evolutionary rate differences among sites were modeled using a discrete Gamma distribution across five categories (+G, parameter = 2.6253), with 44.28% of sites deemed evolutionarily invariant (+I). The analytical procedure encompassed 89 nucleotide sequences with 648 positions in the final dataset.

The VP1 sequences for serotype SAT 2 isolates (*n* = 49) were characterized within topotype VII (Alx-12 [*n* = 2] and Lib-12 [*n* = 2]) lineages, topotype XIII (*n* = 1) and topotype XIV (*n* = 44) (Figure 5). In general, only one SAT 2 lineage was detected in Ethiopia at a time, with topotypes VII (Alx-12 and Lib-12) viruses detected in 2019 and 2022, respectively (Figure 5). The SAT 2 topotype XIII was detected in 2020. The SAT 2 topotype XIV was detected in 2022–2023. The SAT 2 serotype has been historically prevalent in Ethiopia and sequences trace back to virus ancestors circulating at the turn of the 20th century. For the SAT 2 topotype XIV, the ancestral virus dated to 1991; highlighting a three-decade gap in the detection of this topotype between 1991 and 2022. Sequence analysis of these samples did not detect SAT 1, a finding inconsistent with Ag-ELISA results.

These VP1 coding sequences have been deposited in the National Center for Biotechnology Information (NCBI) GenBank database, as detailed in (Supporting Information 1: Table S1). A total of 94 sequences (20 O/EA-3, 7 O/EA-4, 18 A/AFRICA/G-IV, 2 SAT 2/VII/Alx-12, 2SAT 2/VIII/Lib-12, 1 SAT 2/XIII, and 44 SAT 2/XIV) were submitted.

## 4. Discussion

This study yielded 411 samples that were collected across various regions in Ethiopia over a 5-year period, from 2019 to 2023. A high proportion (81.1%, 172/212) tested positive for the FMDV genome with a pan-serotype RT-PCR. These FMDV-positive samples were used to investigate the distribution of FMDV serotypes in the regions studied. Although the RT-PCR has high analytical and diagnostic sensitivity [39], it lacks typing capacity, as was noted in a previous study in Tanzania [40]. Our study using Ag-ELISA showed that 45 (10.9%), 30 (7.3%), 7 (1.7%), and 80 (19.5%) were positive for FMDV serotypes O, A, SAT 1, and SAT 2, respectively. Virus isolation combined with Ag-ELISA for typing showed an efficiency of 82%. 213/411 (51.8%) of the samples tested negative for all FMD serotypes, potentially due to the quality of samples or the stage of clinical signs of the disease when samples were collected.

Different studies have identified at least four FMDV serotypes in Ethiopia (O, A, SAT 1, and SAT 2) [3, 41–44]. An earlier study for samples collected from 2008 to 2019 [19] reported that serotype O was the most prevalent (*n* = 175), followed by serotype A (*n* = 51) and SAT 2 (*n* = 33). Phylogenetic analysis undertaken in this study identified 27 samples of viruses that were characterized within the serotype O EA-3 and EA-4 topotypes. Serotype O was detected in all years of the study apart from 2023 when only four samples were sequenced of serotyped using Ag-ELISA. These findings mirror data reported for previous years [19] and reinforce the importance of serotype O as a circulating serotype in Ethiopia. Close genetic relationships to previously characterized viruses support the idea that these serotype O viruses can be maintained within the country. Furthermore, sequences from neighboring countries (Sudan, Egypt, Eritrea, and Uganda) show that serotype O viruses from the O/EA-3 can readily pass across international borders (45). The O/EA-3 topotype is widely distributed in Africa and has been reported in Nigeria [45–47], Egypt [35, 48], and Eritrea [49]. Therefore, it is well-documented that O/EA-3 is present in East African countries, including Ethiopia and Sudan, while serotype O/EA-4 is more restricted to Ethiopia [18, 19, 50, 51].

In our study, Ag-ELISA serotyping revealed results consistent with mixed infections, particularly high among SAT 2, and SAT 1 serotypes. A similar study by Tesfaye et al. [43] reported mixed infections; however, previous FMD surveillance studies in Ethiopia show that SAT 1 has not been confirmed in the country since 2007. The initial positive results for SAT1 from the ELISA were ultimately considered as false positives. This conclusion was reached after subsequent VP1 sequencing, a more specific test, failed to detect the SAT1 genome in those same samples. This discrepancy was further supported by the ELISA's own data, as the samples' OD values (ranging from 0.1 to 0.19) were at the marginal end of the 0.1 cut-off, indicating a very weak positive signal that was likely not a true infection.

All of the serotype A viruses (*n* = 18) that were characterized in this study belonged to the A/AFRICA/G-IV genotype. This is a more restricted range of sequences compared to the findings of Gizaw et al. [19], who, in their earlier (2008–2019) study, detected A/AFRICA/G-I, A/AFRICA/G-IV, and A/AFRICA/G-VII lineages in the country. These differences might reflect temporal or regional variations in the circulating serotype A lineages or reflect the ad-hoc samples and biases in the sample collection. Phylogenetic analysis of the FMDV A/AFRICA/G-IV lineage from our study reveals its closest relationship to FMDV isolates previously reported in Ethiopia and Sudan. It is understood that this lineage has established itself in Ethiopia, as other lineages have not been detected for at least the last 5 years. Consistent with our study, Woldemariyam et al. [23] also identified six FMDV lineages classified as serotype A/AFRICA/G-IV in their research conducted in Ethiopia between 2018 and 2020. Furthermore, Tesfaye et al. [43] reported the identification of FMDV A/AFRICA/G-IV in Ethiopia in their 2018 study.

Serotype SAT 2 was the most common serotype that was monitored in this study. Sequence analyses revealed the circulation of diverse SAT 2 lineages across the country, including SAT 2 topotype VII (Alx-12 and Lib-12), topotype XIII, and topotype XIV. Over the time course of the study, the SAT2/XIV topotype became the most frequent FMDV lineage that was detected. This topotype has recently reappeared after its first and only previous identification in 1991 represented by two isolates (ETH/2/91 and ETH/3/91) reported by Sahle et al. [20]. The re-emergence of the FMDV SAT 2 virus in Ethiopia is a critical issue. While the exact reasons for its period of nondetection are not fully understood, it is believed that SAT2/XIV viruses may have persisted in a wildlife reservoir such as the African buffalo, which are more difficult to monitor. This situation was likely compounded by limited surveillance in remote areas and a lack of dedicated investigation into wildlife populations. The uncontrolled movement of livestock, a common practice in the country, also plays a major role in the virus's spread and maintenance, making it difficult to contain and track. This topotype has caused outbreaks in several Western Asian countries, including Bahrain, Iraq, Jordan, Oman, and Türkiye [52], where sequences point to a likely origin in EA [52] which has been introduced via the trade in animals. Similar to the current study, Gizaw et al. [19] reported diverse SAT/VII and SAT 2/XIII in a study carried out in Ethiopia from 2008 to 2019.

While our current study provides valuable insights into the circulating FMDV serotypes and lineages, it is important to acknowledge its limitations. Notably, we were unable to sequence all FMDVs detected through RT-PCR and antigen-detection ELISA. Consequently, the outbreak investigation presented here represents a snapshot in time and may not fully capture the complete spectrum of FMDV lineages currently circulating within the country due to the inherent constraints of our investigation. Molecular analysis did not confirm outbreaks despite Ag-ELISA positives for SAT 1. This suggests some Ag-ELISA findings for SAT 1 might not represent true infections, impacting the precision of prevalence estimates. These factors motivate the development and validation of new simple test methods that can be deployed into endemic setting to characterize circulating FMDVs, including lineage-specific RT-PCR assays [53, 54] and LFDs that can serotype FMDVs present in samples [55]. Despite these limitations, our findings underscore the continued importance of serotypes O, SAT 2, and A in the FMDV landscape and the importance of the central and western regions of Ethiopia. Furthermore, this study provides compelling evidence for the significant re-emergence of SAT 2 topotype XIV within the country, a lineage with implications for regional and potentially global FMDV epidemiology. Fluctuations in the distribution of the FMDV serotypes in Ethiopia can be attributed to various factors, which include genetic diversity, host population dynamics, environmental factors, vaccination and immunity, trade and movement of animals, and surveillance and diagnostic capabilities.

## 5. Conclusion

Our study reveals the continued importance of FMDV serotypes (O, SAT 2, and A) in Ethiopia during 2019–2023. The increasing dominance of FMDV SAT 2 serotypes highlighted in our study suggests a potentially important shift in the circulating SAT 2 strains within Ethiopia associated with the re-emergence of the SAT 2 topotype XIV. These trends underscore the evolving FMDV situation in Ethiopia, guiding regional control efforts and vaccine strategies. Further comprehensive studies are needed for a complete national picture. The detection of multiple FMDV serotype lineages (O, A, and SAT 2) in Ethiopia is significant because it provides crucial data for the entire FMDV pool 4 region. These findings not only enhance our understanding of FMDV in Ethiopia, but also have broader implications for regional control strategies and vaccine selection. Given that pool 4 includes other Eastern African countries with similar circulating strains, these results underscore the need for a collaborative approach. By sharing control strategies and coordinating vaccine selection, nations in this pool can implement more effective and sustainable FMD management across the region, which is ultimately more impactful than isolated efforts. Future studies with broader geographical coverage and more extensive sequencing efforts are needed to provide a more comprehensive picture of FMDV circulation in the country.

## Figures and Tables

**Table 1:** Detection of FMDV by antigen detection ELISA and RT-PCR in the samples across the different regions in Ethiopia from 2019 to 2023.

Ethiopian region	Ag-ELISA test			RT-PCR test		
	*n*	Positive samples	Percentage positive	*n*	Positive samples	Percentage positive
Addis Ababa	4	3	75.0	2	2	100
Afar	24	2	8.3	3	3	100
Amhara	34	22	64.7	11	9	81.8
Benishangul Gumuz	14	4	28.6	10	10	100
Oromia	254	124	48.8	151	119	78.8
SNNPR^a^	54	29	53.7	28	23	82.1
Somali	9	7	77.8	3	2	66.7
Tigray	18	7	38.9	4	4	100
Total	411	198	48.2	212	172	81.1

^a^Southern Nations, Nationalities, and People's Regional State.

**Table 2:** Comparative analysis of Ag-ELISA and RT-PCR test results using a 2 x 2 contingency table.

Ag-ELISA result	RT-PCR result		
	Positive	Negative	Total
Positive	132	29	161
Negative	40	11	51
Total	172	40	212

**Table 3:** The spatial distribution of FMDV serotypes detected by Ag-ELISA during outbreak investigations in Ethiopia from 2019 to 2023 (*N* = 411).

Ethiopian region	Ag- ELISA					
	Negative	FMDV O	FMDV A	FMDV SAT 2	FMDV SAT 1	Total
Addis Ababa	1	3	—	—	—	3
Afar	22	—	—	2	—	2
Amhara	12	5 (1)	2 (2)	3 (9)	—	22
Benishangul Gumuz	10	1	—	3	—	4
Oromia	130	24 (6)	23 (2)	56 (7)	6	124
SNNPR^a^	25	5	(2)	14 (7)	1	29
Somali	2	4	3	—	—	7
Tigray	11	3	2	2	—	7
Total	213	45 (7)	30 (6)	80 (23)	7	198

*Note:* The numbers in brackets in the FMDV O and FMDV A columns indicate evidence of mixed infections with serotype SAT 2, while the numbers in the SAT 2 column indicate mixed infections with serotype SAT 1.

^a^Southern Nations, Nationalities, and People's Regional State.

## Data Availability

All data generated or analyzed during this study are included in this published article (and its supporting information files).

## Supporting Information

Supporting Information Table S1: The dataset of foot-and-mouth disease virus sequences accessioned in the NCBI GenBank database from 2019 to 2023. This file contains the complete list of accession numbers, collection dates, and relevant metadata used for the phylogenetic analysis presented in this study.
