# Supplementary material for: Serotype Diversity and Molecular Characterization of Foot-and-Mouth Disease Viruses From Outbreaks in Ethiopia (2019–2023): Re-Emergence of SAT 2 After 30 Years
Source: Transbound Emerg Dis. 2025 Nov 26;2025:6670343. doi: 10.1155/tbed/6670343 (PMC12674864; doi:10.1155/tbed/6670343)
Supplement: Supporting Information — Table S1: The dataset of foot-and-mouth disease virus sequences accessioned in the NCBI GenBank database from 2019 to 2023. This file contains the complete list of accession numbers, collection dates, and relevant metadata used for the phylogenetic analysis presented in this study. [file 6670343.f1.docx]

Supplementary Table S1: The dataset of Foot-and-Mouth Disease virus sequences accessioned in the NCBI GenBank database from 2019 to 2023

| **WRLFMD Ref. No.** | **Serotype** | **Topotype** | **Lineage** | **Accession** | **Date collected** | **Species** | **Region** | **Zone** | **District** |
| --- | --- | --- | --- | --- | --- | --- | --- | --- | --- |
| ETH/4/2019 | O | EA-3 |  | MN987483 | 2/7/2019 | cattle | Tigray | Southern Zone | Korem |
| ETH/5/2019 | O | EA-3 |  | MN987484 | 2/7/2019 | cattle | Tigray | Southern Zone | Raya Alamata |
| ETH/6/2019 | O | EA-3 |  | MN987485 | 2/19/2019 | cattle | Tigray | Eastern zone | Senkata,saidaImba |
| ETH/7/2019 | O | EA-3 |  | MN987486 | 2/19/2019 | cattle | Tigray | Southern Zone | Tekia, ImbaAlaje, |
| ETH/8/2019 | O | EA-3 |  | MN987487 | 2/19/2019 | cattle | Tigray | Southern Zone | Tekia, ImbaAlaje, |
| ETH/9/2019 | O | EA-3 |  | MN987488 | 2/19/2019 | cattle | Tigray | Southern Zone | Ayida, ImbaAlaje |
| ETH/14/2019 | O | EA-4 |  | MN987489 | 2/28/2019 | cattle | SNNPRS | Hadiya | Mololcho, Shoshoge |
| ETH/24/2019 | O | EA-4 |  | PQ847887 | 8/24/2019 | bovine | Amhara | Awi | Guangua |
| ETH/26/2019 | O | EA-4 |  | PQ847888 | 10/4/2019 | bovine | Oromia | Horuguduru | Shambu |
| ETH/27/2019 | O | EA-4 |  | PQ847889 | 10/18/2019 | bovine | Oromia | South West Shoa | Sebeta |
| ETH/28/2019 | O | EA-4 |  | PQ847890 | 10/18/2019 | bovine | Oromia | South West Shoa | Sebeta |
| ETH/29/2019 | O | EA-3 |  | PQ847891 | 11/15/2019 | bovine | Oromia | East Shoa | Adama |
| ETH/2/2020 | O | EA-3 |  | PQ847892 | 1/20/2020 | bovine | Oromia | Borana | Gomole |
| ETH/5/2020 | O | EA-3 |  | PQ847893 | 2/10/2020 | bovine | Oromia | South West Shoa | Alemgena |
| ETH/8/2020 | O | EA-4 |  | PQ847894 | 4/23/2020 | bovine | Oromia | Special zone | Kuyu |
| ETH/10/2020 | O | EA-3 |  | PQ847895 | 5/21/2020 | bovine | SNNPRS | WolaitaSodo | Sodo |
| ETH/11/2020 | O | EA-3 |  | PQ847896 | 5/21/2020 | bovine | SNNPRS | WolaitaSodo | Sodo |
| ETH/12/2020 | O | EA-4 |  | PQ847897 | 10/21/2020 | bovine | Oromia | South West Shoa | Sebeta |
| ETH/15/2020 | O | EA-3 |  | PQ847898 | 12/15/2020 | bovine | Oromia | East Shoa | Hankole |
| ETH/16/2020 | O | EA-3 |  | PQ847899 | 12/15/2020 | bovine | Oromia | East Shoa | Hankole |
| ETH/17/2020 | O | EA-3 |  | PQ847900 | 12/15/2020 | bovine | Oromia | East Shoa | Hankole |
| ETH/1/2021 | O | EA-3 |  | PQ847901 | 1/22/2021 | bovine | Amhara | West Gojam | Chanfer |
| ETH/2/2021 | O | EA-3 |  | PQ847902 | 5/21/2021 | bovine | SNNPRS | WolaitaSodo | DamoteWeyde |
| ETH/4/2021 | O | EA-3 |  | PQ847903 | 9/21/2021 | bovine | Addis Ababa | Bole subcity | Wereda 09 |
| ETH/5/2021 | O | EA-3 |  | PQ847904 | 9/21/2021 | bovine | Addis Ababa | Bole subcity | Wereda 10 |
| ETH/5/2022 | O | EA-3 |  | PQ847905 | 3/30/2022 | bovine | Oromia | North Shoa | DebreLibanose |
| ETH/21/2022 | O | EA-3 |  | PQ847906 | 7/5/2022 | cattle | BenshangulGumz | Assosa | Bombasi mender 47 |
| ETH/1/2019 | A | AFRICA | G-IV | MN987534 | 2/6/2019 | cattle | Oromia | West Shoa | AdeaBerga |
| ETH/2/2019 | A | AFRICA | G-IV | MN987535 | 2/6/2019 | cattle | Oromia | West Shoa | AdeaBerga |
| ETH/3/2019 | A | AFRICA | G-IV | MN987536 | 2/6/2019 | cattle | Oromia | West Shoa | AdeaBerga |
| ETH/11/2019 | A | AFRICA | G-IV | MN987537 | 2/28/2019 | cattle | Oromia | West Shoa | Wolmera |
| ETH/12/2019 | A | AFRICA | G-IV | MN987538 | 2/28/2019 | cattle | Oromia | West Shoa | Wolmera |
| ETH/13/2019 | A | AFRICA | G-IV | MN987539 | 2/28/2019 | cattle | Oromia | West Shoa | Wolmera |
| ETH/16/2019 | A | AFRICA | G-IV | MN987540 | 3/9/2019 | cattle | Oromia | Horuguduru | Kombolcha, Guduru, |
| ETH/18/2019 | A | AFRICA | G-IV | MN987541 | 3/9/2019 | cattle | Oromia | Horuguduru | Keneni |
| ETH/19/2019 | A | AFRICA | G-IV | MN987542 | 3/9/2019 | cattle | Oromia | Horuguduru | Guduru |
| ETH/21/2019 | A | AFRICA | G-IV | PQ847907 | 4/9/2019 | bovine | SNNPRS | South Omo | Bero |
| ETH/25/2019 | A | AFRICA | G-IV | PQ847908 | 8/26/2019 | bovine | Oromia | East Shoa | Lume |
| ETH/30/2019 | A | AFRICA | G-IV | PQ847909 | 11/22/2019 | bovine | Tigray | Southern Zone | Samere |
| ETH/34/2019 | A | AFRICA | G-IV | PQ847910 | 12/20/2019 | bovine | Oromia | South West Shoa | Sebeta |
| ETH/1/2020 | A | AFRICA | G-IV | PQ847911 | 1/15/2020 | bovine | Oromia | Finfinespecialzone | Holeta |
| ETH/9/2020 | A | AFRICA | G-IV | PQ847912 | 5/9/2020 | bovine | Oromia | Horuguduru | Jima Genete |
| ETH/13/2020 | A | AFRICA | G-IV | PQ847913 | 11/11/2020 | bovine | Oromia | Arsi | Monesa |
| ETH/14/2020 | A | AFRICA | G-IV | PQ847914 | 11/11/2020 | bovine | Oromia | Arsi | Monesa |
| ETH/4/2022 | A | AFRICA | G-IV | PQ847915 | 3/30/2022 | bovine | Oromia | North Shoa | DebreLibanose |
| ETH/31/2019 | SAT2 | VII | Lib-12 | PQ847916 | 11/22/2019 | bovine | Tigray | Southern Zone | Mekeric |
| ETH/32/2019 | SAT2 | VII | Lib-12 | PQ847917 | 11/22/2019 | bovine | Tigray | Mekele | Semen Mekele |
| ETH/9/2020 | SAT2 | XIII |  | PQ847918 | 5/9/2020 | bovine | Oromia | Horuguduru | Jima Genete |
| ETH/2/2022 | SAT2 | XIV |  | PQ847919 | 3/29/2022 | bovine | SNNPRS | WolaitaSodo | Sodo |
| ETH/3/2022 | SAT2 | XIV |  | PQ847920 | 3/29/2022 | bovine | SNNPRS | WolaitaSodo | Sodo |
| ETH/9/2022 | SAT2 | XIV |  | PQ847921 | 5/28/2022 | cattle | SNNPRS | Hadiya | Gibe |
| ETH/10/2022 | SAT2 | XIV |  | PQ847922 | 5/28/2022 | cattle | SNNPRS | Hadiya | Gibe |
| ETH/11/2022 | SAT2 | XIV |  | PQ847923 | 5/31/2022 | cattle | Oromia | Jima | Dedo |
| ETH/110/2022 | SAT2 | XIV |  | PQ847958 | 12/8/2022 | cattle | Oromia | South West Shoa | Sebeta |
| ETH/111/2022 | SAT2 | XIV |  | PQ847959 | 12/8/2022 | cattle | Oromia | South West Shoa | Sebeta |
| ETH/112/2022 | SAT2 | XIV |  | PQ847960 | 12/8/2022 | cattle | Oromia | South West Shoa | Sebeta |
| ETH/12/2022 | SAT2 | XIV |  | PQ847924 | 5/31/2022 | cattle | Oromia | Jima | Dedo |
| ETH/13/2022 | SAT2 | XIV |  | PQ847925 | 5/31/2022 | cattle | Oromia | Jima | Dedo |
| ETH/16/2022 | SAT2 | XIV |  | PQ847926 | 5/31/2022 | cattle | Oromia | South West Shoa | Goro |
| ETH/17/2022 | SAT2 | XIV |  | PQ847927 | 5/31/2022 | cattle | Oromia | South West Shoa | Goro |
| ETH/20/2022 | SAT2 | XIV |  | PQ847928 | 7/5/2022 | cattle | Oromia | North Shoa | Sheno |
| ETH/26/2022 | SAT2 | VII | Alx-12 | PQ847929 | 9/9/2022 | cattle | Afar | Kilelu | Gawane |
| ETH/27/2022 | SAT2 | VII | Alx-12 | PQ847930 | 9/9/2022 | cattle | Afar | Kilelu | Gawane |
| ETH/28/2022 | SAT2 | XIV |  | PQ847931 | 9/14/2022 | cattle | Oromia | North Shoa | Degam |
| ETH/29/2022 | SAT2 | XIV |  | PQ847932 | 9/14/2022 | cattle | Oromia | North Shoa | Degam |
| ETH/31/2022 | SAT2 | XIV |  | PQ847933 | 9/14/2022 | cattle | Oromia | North Shoa | Degam |
| ETH/34/2022 | SAT2 | XIV |  | PQ847934 | 9/14/2022 | cattle | Oromia | North Shoa | Degam |
| ETH/43/2022 | SAT2 | XIV |  | PQ847935 | 10/5/2022 | cattle | Oromia | Borana | Arero |
| ETH/44/2022 | SAT2 | XIV |  | PQ847936 | 10/5/2022 | cattle | Oromia | Borana | Arero |
| ETH/47/2022 | SAT2 | XIV |  | PQ847937 | 10/22/2022 | cattle | Oromia | East Shoa | Gidukombolcha |
| ETH/48/2022 | SAT2 | XIV |  | PQ847938 | 10/22/2022 | cattle | Oromia | East Shoa | Gidukombolcha |
| ETH/49/2022 | SAT2 | XIV |  | PQ847939 | 10/22/2022 | cattle | Oromia | East Shoa | Gidukombolcha |
| ETH/50/2022 | SAT2 | XIV |  | PQ847940 | 10/22/2022 | cattle | Oromia | East Shoa | Gidukombolcha |
| ETH/51/2022 | SAT2 | XIV |  | PQ847941 | 10/22/2022 | cattle | Oromia | East Shoa | Gidukombolcha |
| ETH/57/2022 | SAT2 | XIV |  | PQ847942 | 10/22/2022 | cattle | Oromia | East Shoa | Alemtena |
| ETH/61/2022 | SAT2 | XIV |  | PQ847943 | 10/22/2022 | cattle | Oromia | East Shoa | Alemtena |
| ETH/62/2022 | SAT2 | XIV |  | PQ847944 | 10/22/2022 | cattle | Oromia | East Shoa | Alemtena |
| ETH/63/2022 | SAT2 | XIV |  | PQ847945 | 10/22/2022 | cattle | Oromia | East Shoa | Alemtena |
| ETH/64/2022 | SAT2 | XIV |  | PQ847946 | 10/22/2022 | cattle | Oromia | East Shoa | Alemtena |
| ETH/66/2022 | SAT2 | XIV |  | PQ847947 | 10/22/2022 | cattle | Oromia | East Shoa | Alemtena |
| ETH/75/2022a | SAT2 | XIV |  | PQ847948 | 10/24/2022 | cattle | Oromia | West Arsi | NegeleArsi |
| ETH/75/2022b | SAT2 | XIV |  | PQ847949 | 10/24/2022 | cattle | Oromia | West Arsi | NegeleArsi |
| ETH/77/2022 | SAT2 | XIV |  | PQ847950 | 10/24/2022 | cattle | Oromia | West Arsi | NegeleArsi |
| ETH/84/2022 | SAT2 | XIV |  | PQ847951 | 11/6/2022 | cattle | Oromia | West Arsi | Shashamene |
| ETH/90/2022 | SAT2 | XIV |  | PQ847952 | 11/14/2022 | cattle | Oromia | South West Shoa | SedenSodo |
| ETH/105/2022 | SAT2 | XIV |  | PQ847953 | 12/8/2022 | cattle | Oromia | South West Shoa | Sebeta |
| ETH/106/2022 | SAT2 | XIV |  | PQ847954 | 12/8/2022 | cattle | Oromia | South West Shoa | Sebeta |
| ETH/107/2022 | SAT2 | XIV |  | PQ847955 | 12/8/2022 | cattle | Oromia | South West Shoa | Sebeta |
| ETH/108/2022 | SAT2 | XIV |  | PQ847956 | 12/8/2022 | cattle | Oromia | South West Shoa | Sebeta |
| ETH/109/2022 | SAT2 | XIV |  | PQ847957 | 12/8/2022 | cattle | Oromia | South West Shoa | Sebeta |
| ETH/1/2023 | SAT2 | XIV |  | PQ847961 | 1/1/2023 | cattle | Oromia | Arsi | Digaluenatijo |
| ETH/2/2023 | SAT2 | XIV |  | PQ847962 | 1/1/2023 | cattle | Oromia | Arsi | Digaluenatijo |
| ETH/3/2023 | SAT2 | XIV |  | PQ847963 | 1/1/2023 | cattle | Oromia | Arsi | Digaluenatijo |
| ETH/4/2023 | SAT2 | XIV |  | PQ847964 | 1/1/2023 | cattle | Oromia | Arsi | Digaluenatijo |
